# Risk and predictors of adverse pathology after radical prostatectomy in patients diagnosed with IUSP 1–2 prostate cancer at MRI-targeted biopsy: a multicenter analysis

**DOI:** 10.1007/s00345-022-04236-4

**Published:** 2022-12-19

**Authors:** Claudia Kesch, Vlad Pantea, Timo Soeterik, Alessandro Marquis, Giulia la Bombarda, Allesandro Morlacco, Francesco Barletta, Jan Philipp Radtke, Christopher Darr, Felix Preisser, Fabio Zattoni, Giancarlo Marra, Roderik C. N. van den Bergh, Boris Hadaschik, Giorgio Gandaglia, On Behalf of the EAU-YAU Prostate Cancer Working Party

**Affiliations:** 1 Department of Urology, University Hospital Essen, Hufelandstrasse 55, 45147 Essen, Germany; 2 Department of Urology, St. Antonius Hospital, Nieuwegein-Utrecht, The Netherlands; 3 Department of Urology, San Giovanni Battista Hospital, Città della Salute e della Scienza and University of Turin, Turin, Italy; 4 Department of Surgery, Oncology and Gastroenterology, Urology Clinic, University of Padova, Padua, Italy; 5 Division of Oncology/Unit of Urology, Urological Research Institute, IRCCS Ospedale San Raffaele, Milan, Italy; 6 Department of Urology, University Hospital Frankfurt, Frankfurt, Germany

**Keywords:** Prostate cancer, Radical prostatectomy, Active surveillance, Outcomes, ISUP

## Abstract

**Purpose:**

Although active surveillance (AS) is recommended for low- to favorable intermediate-risk prostate cancer (PCa), risk of upgrading at radical prostatectomy (RP) is not negligible. Available studies based on systematic transrectal ultrasound biopsy might not be applicable to contemporary cohorts diagnosed with MRI-targeted biopsy (TB). The aim of the present study is to explore rates and risk factors for adverse outcomes (AO) at RP in patients with ISUP ≤ 2 PCa detected at TB with concomitant systematic biopsy (SB).

**Methods:**

Multicenter, retrospective analysis of 475 consecutive patients with ISUP ≤ 2 PCa at MRI-TB + SB is treated with RP. AO were defined as ISUP upgrading, adverse pathology (upgrading to ISUP ≥ 3 and/or ≥ pT3 at RP, and/or pN1) (AP) or biochemical recurrence (BCR) in men with follow-up (*n* = 327).

**Results:**

The rate of ISUP upgrading, upgrading ≥ 3, and AP were 39%, 21%, and 43%. Compared to ISUP2, men with ISUP1 PCa had a higher rate of overall upgrading (27 vs. 67%, *p* < 0.001), but less upgrading to ≥ 3 (27 vs. 10%, *p* < 0.001). AP was more common when ISUP2 was detected with a combined MRI-TB + SB approach compared to considering TB (*p* = 0.02) or SB (*p* = 0.01) alone. PSA, PSA density, PI-RADS, ISUP at TB, overall biopsy ISUP and EAU classification were predictors of upgrading to ISUP ≥ 3 and AP. The 1 year BCR-free survival was 94% with no differences in BCR rates between subgroups.

**Conclusion:**

Upgrading in ISUP ≤ 2 PCa remains prevalent even in men diagnosed in the MRI era. The use of MRI-TB with concomitant SB allows for the accurate identification of ISUP2 PCa and predicts the risk of AO at RP.

**Supplementary Information:**

The online version contains supplementary material available at 10.1007/s00345-022-04236-4.

## Introduction

Since the introduction of PSA [1], overdiagnosis and overtreatment represent main issues in prostate cancer (PCa) management. Multiparametric magnetic resonance imaging (mpMRI) has been introduced in the clinical practice [2–4] and can be used as a triage test to identify men with clinical suspicion of PCa who should receive a prostate biopsy. The routine use of mpMRI in the diagnostic pathway of PCa might allow for the identification of aggressive disease requiring radical therapy and theoretically miss indolent cancers who could ideally be considered for active surveillance (AS). Surveillance has been widely accepted as recommended treatment for low-risk PCa [5] with equivalent oncological outcomes compared to definitive therapy at medium-term follow-up [6, 7]. Moreover, more than half of the established AS protocols allow for the inclusion of patients with intermediate-risk features [8]. Nonetheless, the risk of unfavorable pathology at radical prostatectomy (RP) is substantials up to 78% AS candidates managed with RP harbored adverse disease features at final pathology [9]. However, these observations derive from cohorts diagnosed with random transrectal ultrasound (TRUS) biopsy. Due to the higher accuracy for the identification of clinically significant PCa by MRI-guided biopsy [2–4], the generalizability of these findings in the mpMRI era is not warranted.

We hypothesized that mpMRI and MRI-targeted biopsy would allow for the reliable identification of AS candidates thus resulting in a lower risk of upgrading and adverse pathology (AP) in low- and favorable intermediate-risk patients undergoing RP. We aimed at reporting the rate and at identifying predictors of upgrading and early biochemical recurrence (BCR) in men with mpMRI/TRUS-fusion biopsy diagnosed ISUP 1 and 2 PCa and managed with RP.

## Patients and methods

### Study population

Retrospective databases from seven European tertiary referral centers were taken. Board approval was obtained according to each institution’s policy. We identified 475 patients that underwent RP for ISUP ≤ 2 PCa on mpMRI/TRUS-fusion biopsy between the years 2016 and 2021. mpMRIs were performed according to each institutions protocol and scored by expert genitourinary radiologists using PI-RADS v2 [10]. MRI-targeted fusion biopsy (TB) with concomitant systematic (SB) was performed by experienced urologists using their preferred biopsy technique including cognitive or software supported biopsy. All patients were treated with robot-assisted or open RP. Histopathology was reviewed in each center according to clinical routine by expert dedicated urogenital pathologist. Follow-up data were available for 327 patients. Data were reported according to the Standards of Reporting for MRI-Targeted Biopsy Studies (START) [11].

### Outcome measurements and predictors

Adverse outcomes (AO) were defined as ISUP upgrading or adverse pathology (AP = upgrading to ISUP ≥ 3 and/or ≥ pT3 at RP, and/or N1). BCR was defined as two consecutive PSA values ≥ 0.2 ng/mL. Patients were stratified according to EAU low-risk and favorable intermediate-risk (ISUP 2, PSA < 10 ng/ml and ≤ T2).

Age, digital rectal examination (DRE), prostate volume, PSA, PSA-Density, PI-RADS, targeted lesion volume, extracapsular extension (ECE) on mpMRI, number of TB cores, percentage of positive TB cores, ISUP at TB, number of SB cores, percentage of positive SB cores, ISUP at SB, number of overall cores, percentage of positive cores, overall ISUP and EAU classification group were analyzed as variables predicting AO and all of the above plus ISUP at RP, ISUP upgrading, upgrading to ISUP ≥ 3, a ≥ pT3, any AO and positive surgical margins were analyzed as variables predicting BCR.

### Statistical analysis

Differences between groups were assessed by using chi-square test for categorial and *T* test for continuous variables. Logistic and COX regression analyses determined predictive factors. Due to the relatively small number of events, univariable analyses were used to assess predictors of outcomes. Results were considered statistically significant for *p* < 0.05. Analyses were conducted using SPSS version 27 (IBM, Armonk, NY, USA).

## Results

### Baseline patient characteristics

Baseline patient demographics and clinical characteristics for all patients and stratified to subgroups are shown in Table 1.

**Table 1:** Patient demographics, radical prostatectomy outcomes and recurrence

	Low-risk PCa*	Favorable intermediate-risk PCa”		All PCa
	(*n* = 112)	(*n* = 256)		(*n* = 475)
Median age (range)—year	63 (50–78)	66 (47–79)	*p* = 0.03	66 (45–80)
Biopsy			*p* = 0.80	
Primary biopsy—no. (%)	77 (69)	171 (67)		312 (66)
Repeat biopsy—no. (%)	16 (14)	52 (20)		101 (21)
Under active surveillance —no. (%)	19 (17)	33 (13)		62 (13)
Biopsy route			*p* = 0.01	
Transperineal—no. (%)	20 (18)	88 (34)		139 (29)
Transrectal—no. (%)	92 (82)	168 (66)		336 (71)
mpMRI fusion			*p* = 0.02	
Software based	86 (77)	221 (86)		383 (81)
Cognitive	26 (23)	35 (14)		92 (19)
Positive DRE—no. (%)	29 (26)	81 (32)	*p* = 0.26	141 (30)
Median prostate volume (range)—ml^#^	45 (16–136)	43 (8–202)	*p* = 0.06	45 (8–202)
Median PSA (range)—ng/ml	6 (1–10)	6 (1–10)	*p* = 0.35	6 (1–47)
Median density (range)—ng/ml/ml^#^	0.130 (0.010–0.360)	0.130 (0.010–0.440)	*p* = 0.28	0.150 (0.010–1.290)
Overall PI-RADS			*p* = 0.06	
1—no. (%)	0 (0)	1 (0)		1 (0)
2—no. (%)	7 (6)	11 (4)		22 (5)
3—no. (%)	30 (27)	39 (15)		88 (18)
4—no. (%)	55 (49)	152 (60)		247 (52)
5—no. (%)	20 (18)	53 (21)		117 (25)
Median mpMRI visible lesions (range)—no	1 (0–4)	1 (0–4)	*p* = 0.73	1 (0–4)
Median combined lesion volume (range)—ml^§^	1.77 (0.03–24.42)	0.57 (0.01–28.26)	*p* = 0.02	0.86 (0.01–38.77)
Extraprostatic extension on mpMRI—no. (%)	12 (11)	38 (15)	*p* = 0.26	71 (15)
Median targeted biopsies (range)—no	3 (0–20)	4 (0–18)	*p* = 0.20	3 (0–20)
Median positive targeted biopsies (range)—no	1 (0–7)	2 (0–11)	*p* < 0.01	2 (0–11)
Median systematic biopsies (range)—no	12 (0–28)	12 (3–24)	*p* < 0.01	12 (0–28)
Median positive systematic biopsies (range)—no	2 (0–11)	3 (0–12)	*p* = 0.98	3 (0–12)
Overall biopsy ISUP				
1—no. (%)	112 (100)	0 (0)		147 (31)
2—no. (%)	0 (0)	256 (100)		328 (69)
RP ISUP			*p* < 0.01	
1—no. (%)	39 (35)	8 (3)		58 (12)
2—no. (%)	68 (61)	187 (73)		316 (67)
3—no. (%)	4 (4)	55 (22)		89 (19)
4—no. (%)	1 (0)	6 (2)		8 (2)
5—no. (%)	0 (0)	0 (0)		4 (1)
RP T-stadium			*p* = 0.02	
pT2—no. (%)	87 (78)	174 (68)		324 (68)
pT3—no. (%)	25 (22)	82 (32)		151 (32)
RP positive surgical margins—no. (%)	25 (22)	49 (19)	*p* = 0.08	110 (23,2)
RP positive lymph nodes pN1—no. (%)	0 (0)	3 (1)	*p* = 0.28	7 (1,5)
Any upgrading—no. (%)	73 (65)	61 (24)	*p* < 0.01	185 (39)
Upgrading to ISUP ≥ 3—no. (%)	5 (4)	61 (24)	*p* < 0.01	101 (21)
Adverse pathology	28 (25)	116 (45)	p < 0.01	205 (43,2)
	(*n* = 78)	(*n* = 181)		(*n* = 327)
Median follow-up (range)—month	11 (1–64)	14 (1–62)	*p* = 0.29	12 (1–64)
Biochemial recurrence—no. (%)	3 (4)	11 (4)	*p* = 0.47	20 (6)

*PCa* prostate cancer, *PSA* prostate-specific antigen, *DRE* digital rectal examination, *mpMRI* multiparametric magnet resonance imaging, *ISUP* International Society of Urological Pathology, *RP* radical prostatectomy

*According to EAU, “ISUP 2 and PSA < 10 ng/ml and cT ≤ 2

^#^Data missing from 22 patients

^§^Data missing from 266 patients

### Incidence of adverse outcomes characterized by biopsy approach

**Fig. 1:**
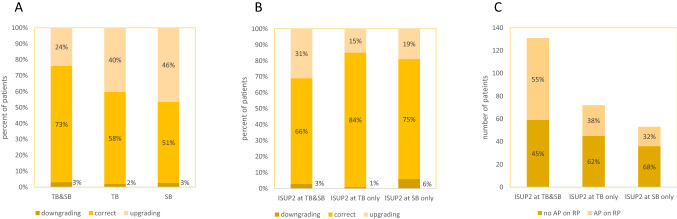
Radical prostatectomy outcomes of patients with favorable intermediate-risk disease. **A** Upgrading and downgrading stratified by biopsy approach; **B** Upgrading and downgrading based on ISUP 2 detecting biopsy approach (ISUP 2 at TB&SB *n* = 131, ISUP 2 at TB only *n* = 72, ISUP 2 at SB only *n* = 53), **C** Adverse pathology findings based on ISUP 2 detecting biopsy approach (ISUP 2 at TB&SB *n* = 131, ISUP 2 at TB only *n* = 72, ISUP 2 at SB only *n* = 53), *TB* targeted biopsies, *SB* systematic biopsies, *ISUP* International Society of Urological Pathology, *AP* adverse pathology, *RP* radical prostatectomy

Analyzing the combined SB and TB approach, 185 (39%) and 101 (21%) patients experienced any ISUP upgrading and upgrading to ISUP ≥ 3, respectively. Biopsy ISUP 1 was upgraded in 98 (67%) patients, but only in 14 (10%) to ISUP ≥ 3. Biopsy ISUP 2 was upgraded in 87 (27%) patients and downgraded in 9 (3%) patients (Supplementary Table 1A). The combined biopsy approach correctly identified 84% of ISUP 1 PCa at RP, which would not have been different using a SB only approach identifying 81% of ISUP 1 PCa (*p* = 0.6). A TB only approach on the other hand would have correctly predicted significantly less ISUP 1 PCa (*p* < 0.001). Eighteen men without cancer at TB harbored ISUP ≥ 3 cancers. Compared to ISUP at RP, the combined approach correctly characterized 73% of ISUP 2 PCa, which is significantly more (*p* < 0.001) than either SB with 52% or TB with 58% alone would have detected (SB vs. TB: *p* = 0.1, Supplementary Table 1). Similar detection rates were found for favorable intermediate-risk only Fig. 1A, where the combined biopsy approach detected significantly more ISUP 2 PCa than TB or SB alone (*p* = 0.001 and *p* < 0.001, respectively).

Compared to the RP specimen, the least upgrading was found when ISUP 2 was present in TB only. Patients with ISUP 2 at TB only had a significantly lower upgrading rate (15%) compared to ISUP 2 at TB and SB (31%) (*p* = 0.01). No significant differences were found comparing upgrading rates of ISUP 2 at SB only (19%) to ISUP 2 at TB and SB (*p* = 0.1) or ISUP 2 at TB only (*p* = 0.555) Fig. 1B. Similarly, men with favorable intermediate-risk disease demonstrated significantly more AP on RP when ISUP 2 features were present in both TB and SB compared to the presence of ISUP 2 features in TB (*p* = 0.021) or SB (*p* = 0.005) only Fig. 1C. In men diagnosed with low-risk Pca, there was significantly less upgrading and less AP if ISUP 1 was only present in SB and not in both SB and TB (*p* = 0.008 and *p* 0.024) Supplementary Fig. 1.

### Incidence of adverse outcomes and biochemical recurrence stratified by subgroups

RP outcomes and incidences of AO and BCR are shown in Table 1. Between the low-risk and the favorable intermediate-risk group, there were significant differences regarding ISUP at RP, RP pT, ISUP upgrading and AP. With 65% overall, including upgrading to ISUP 2, upgrading rates were considerably higher in the low-risk group compared to the favorable intermediate-risk group with 24%. For the 327 patients with available follow-up, the 1 year BCR-free survival was 94%. Neither time of follow-up nor BCR rate did significantly differ between the subgroups.

### Predictors of adverse outcomes and biochemical recurrence

Predictors of AO and BCR are shown in Table 2. Higher PSA, higher PSA density, PI-RADS, ISUP at TB, overall biopsy ISUP and EAU classification were all predictors for upgrading to ISUP ≥ 3 and AP. Additionally, number of SB and overall biopsies and ISUP at SB were predictive for upgrading to ISUP ≥ 3. For pT3, percentage of positive overall biopsies, SBs and TBs were positive predictors and number of overall biopsies and SBs were negative predictors, too. For AP, ISUP at SB and percentage of positive overall and TBs were predictive in addition to the variables described above. None of the primary mpMRI/TRUS-fusion biopsy variables, but all AOs including any ISUP upgrading, upgrading to ISUP ≥ 3, adverse pT (≥ pT3) at RP and AP as well as ISUP at RP were predictors for BCR. Predictors of AO for the subgroups are shown in Supplementary Table 2.

**Table 2:** Predictors for upgrading to ISUP ≥ 3, adverse pathology and BCR (univariate logistic regression)

ISUP upgrading ≥ 3 (*n* = 101)					
PSA	OR	1.04	95%CI	1.01–1.08	*p* = 0.01
PSA density	OR	4.20	95%CI	1.17–15.11	*p* = 0.03
PI-RADS	OR	1.39	95%CI	1.04–1.87	*p* = 0.03
ISUP TB	OR	1.47	95%CI	1.10–1.96	*p* = 0.01
N cores SB	OR	1.05	95%CI	1.01–1.08	*p* = 0.01
ISUP SB	OR	1.66	95%CI	1.17–2.34	*p* = 0.01
N cores overall	OR	1.04	95%CI	1.01–1.07	*p* = 0.01
ISUP All	OR	3.43	95%CI	1.88–6.27	*p* ≤ 0.01
EAU classification	OR	2.68	95%CI	1.69–4.26	*p* ≤ 0.01

AP (*n* = 205)					
PSA	OR	1.04	95%CI	1.01–1.08	*p* = 0.01
PSA density	OR	5.76	95%CI	1.56–21.24	*p* = 0.02
PI-RADS	OR	1.35	95%CI	1.07–1.71	*p* = 0.01
% TB+	OR	1.01	95%CI	1.00–1.01	*p* ≤ 0.01
ISUP TB	OR	1.51	95%CI	1.20–1.90	*p* ≤ 0.01
ISUP SB	OR	1.40	95%CI	1.08–1.83	*p* = 0.01
% overall+	OR	1.01	95%CI	1.00–1.02	*p* ≤ 0.01
ISUP All	OR	2.07	95%CI	1.37–3.11	*p* ≤ 0.01
EAU classification	OR	2.24	95%CI	1.54–3.25	*p* ≤ 0.01

Biochemical recurrence (*n* = 20/327)					
pT ≥ 3	HR	6.58	95%CI	2.35–18.41	*p* ≤ 0.01
ISUP RP	HR	2.36	95%CI	1.49–3.76	*p* ≤ 0.01
ISUP upgrading	HR	3.25	95%CI	1.24–8.49	*p* = 0.02
ISUP upgrading to ≥ 3	HR	3.26	95%CI	1.34–7.94	*p* = 0.01
AP	HR	3.95	95%CI	1.42–11.02	*p* = 0.01

*PSA* prostate-specific antigen, *TB* targeted biopsies, *SB* systematic biopsies, *ISUP* International Society of Urological Pathology, *AP* adverse pathology, *RP* radical prostatectomy

## Discussion

In this multicenter cohort 39% of men with MRI/TRUS-fusion diagnosed ISUP ≤ 2 PCa were upgraded on RP and 21% harbored disease of ISUP ≥ 3. Higher PSA, higher PSA density, PI-RADS, ISUP at TB, overall biopsy ISUP and EAU classification were all predictors for upgrading to ISUP ≥ 3, pT ≥ 3 and AP. The 1 year BCR-free survival was 94% and did not differ between low-risk and favorable intermediate-risk. Any ISUP upgrading, upgrading to ISUP ≥ 3, pT ≥ 3 at RP and AP as well as ISUP at RP were predictors for BCR.

Despite the modern MRI/TRUS-fusion technique used for diagnosis, more than 1 in 5 patients were upgraded to ISUP ≥ 3 and 4 out of 10 patients demonstrated AP on RP. Although we initially hypothesized that the introduction of MRI and TB may result in a better preoperative characterization, these findings highlight that the risk of harboring an ISUP 3 disease with poorer prognosis is still relevant in contemporary patients who could have been considered for AS [12]. Of note, our study represents the first assessment of AP and BCR in a large cohort diagnosed with MRI-targeted biopsy and undergoing RP. As such, comparing our results to cohorts not relying on MRI/TRUS-fusion is complex due to heterogenous populations. Moreover, our data are too immature to draw any final conclusions regarding the correlation of AP and long-term outcomes like metastatic progression and survival.

In a large population-based cohort of patients treated in the 2010s, upgrading was seen in 43–61% of biopsy GS 6 [13]. Schiffmann et al. evaluated more than 1000 patients with very low-risk and low-risk disease and found upgrading rates of 55% and 78%, respectively [9]. Other studies focused on patients with favorable intermediate-risk PCa. In a series of over 10,000 patients, Yang et al. found a 33% incidence of upgrading or upstaging [14]. One study including intermediate-risk disease has found AP outcomes in 36% of patients [15]. However, in men with biopsy GS 3 + 4 and cT1–2 stage, Ploussard et al. reported that the rate of GS upgrading was 25% [16]. In another cohort including low- and intermediate-risk patients, Gandaglia et al. reported 33% of patients with unfavorable disease defined as non-organ confined or ISUP ≥ 3 disease at RP [17].

Our data support recent evidence suggesting that compared to an SB approach upfront MRI, an TB detect equal or even more intermediate-risk PCa, while decreasing the detection rate of low-risk PCa [3, 18–20]. However, we also show that compared to the combined approach, a TB only approach would have characterized significantly less ISUP ≥ 2 PCa correctly. This adds to the debate on whether TB alone is sufficient to diagnose higher grade PCa accurately or whether additional SB is still necessary. In the favorable intermediate-risk subgroup, ISUP 2 disease diagnosed at TB only was most concordant to RP and had a significantly lower risk of upgrading compared to ISUP 2 detected both at TB and SB. Men with IUSP 2 features on both SB and TB had a higher likelihood of experiencing AP compared to ISUP 2 features in either TB or SB alone. To the best of our knowledge, this is the first time demonstrating that upgrading and AP in favorable intermediate-risk disease depends on the biopsy approach detecting ISUP 2 features. Based on this, considering patients with favorable intermediate-risk PCa for AS seems more justifiable when the diagnosis was made by a combined biopsy approach and ISUP 2 features are limited to a small amount of positive cores in TB or SB only. Further strategies to improve TB performance are currently evaluated and have already been shown to increase the accuracy of higher grade PCa detection. These include extending the number of TB and target saturation biopsies including the perilesional penumbra [19, 21–23].

Previous studies identified multiple factors for upgrading and AO at RP, including but not limited to age, PSA, cT, number and percentage of positive cores, tumor involvement per core, and perineural invasion [9, 13–17, 24]. Although we describe similar predictors, some findings should be further discussed. PI-RADS was identified as a predictor for upgrading to ISUP ≥ 3 and AP as previously reported in a single-institution study by Pham et al. [25]. Moreover, ECE on mpMRI predicted AP in the subgroup with low-risk PCa and lesion volume predicted upgrading to ISUP ≥ 3 in the subgroup with favorable intermediate-risk PCa. This emphasizes the role mpMRI findings should play when counseling our patients. Indeed, patients eligible for AS were more likely to experience upgrading at RP when suspect lesions were identified on mpMRI compared to unsuspicious mpMRIs [26–28]. The fact that a rising number of SB and overall biopsies is predictive for upgrading to ISUP ≥ 3 seems counterintuitive. This could be explained with a sampling error oversampling Gleason 3 pattern and non-cancerous tissue with extensive systematic biopsies. To fully address this question, histopathology result and sampling location of each individual core would need to be analyzed. At present, our data confirm the updated EAU guidelines position, that saturation biopsy is not being significantly more conclusive than 10–12 cores.

Despite being the first study assessing rate and predictors of AP and BCR in the MRI-targeted biopsy era, our study is not devoid of limitations. There is a selection bias, as information on which factors influenced physicians’ and patients’ decision toward RP is missing. There was no central radiology or pathology review and variations in the interpretation of findings might have influenced the results. No distinction was made between transrectal and perineal as well as cognitive and software-based fusion biopsy. Some information on known predictors of unfavorable outcomes like PCa core percentage or perineural invasion were not available for all institutions and therefore not incorporated into the analysis. Last, the study is subject to the usual limitations of retrospective studies.

## Conclusions

Pathological upgrading in patients diagnosed with ISUP ≤ 2 PCa remains prevalent in the era of MRI/TRUS-fusion biopsy. Although a TB only approach reduces the diagnosis of low-risk PCa, a combined SB and TB approach more accurately characterizes ISUP 2 and risk of AP at RP. Thus, AS for patients with favorable intermediate-risk PCa seems more justifiable when the diagnosis was made by a combined biopsy approach and ISUP 2 features are limited to a small amount of positive cores in TB or SB, only.

## Supplementary Information

Below is the link to the electronic supplementary material.

Supplementary file1 Radical prostatectomy outcomes of patients with low-risk disease. **B** Upgrading and downgrading based on ISUP 1 detecting biopsy approach (ISUP 1 at TB&SB *n* = 56, ISUP 1 at TB only *n* = 13, ISUP 1 at SB only *n* = 43) **C** Adverse pathology findings based on ISUP 2 detecting biopsy approach (ISUP 1 at TB&SB *n* = 56, ISUP 1 at TB only *n* = 13, ISUP 1 at SB only *n* = 43), *TB* targeted biopsies, *SB* systematic biopsies, *ISUP* International Society of Urological Pathology, *AP* adverse pathology, *RP* radical prostatectomy (PPTX 877 KB)

Supplementary file2 ISUP upgrading from biopsy to radical prostatectomy (DOCX 15 KB)

Supplementary file3 Predictors for upgrading to ISUP ≥ 3 and adverse pathology stratified by subgroups (univariate logistic regression) (DOCX 17 KB)

## Data Availability

The data that support the findings of this study are available from the corresponding author, Claudia Kesch, upon reasonable request.
