# Supplementary material for: Risk and predictors of adverse pathology after radical prostatectomy in patients diagnosed with IUSP 1–2 prostate cancer at MRI-targeted biopsy: a multicenter analysis
Source: World J Urol. 2022 Dec 19;41(2):427–34. doi: 10.1007/s00345-022-04236-4 (PMC9947075; doi:10.1007/s00345-022-04236-4)
Supplement: Supplementary file 1 — Supplementary file1 Radical prostatectomy outcomes of patients with low-risk disease. B Upgrading and downgrading based on ISUP 1 detecting biopsy approach (ISUP 1 at TB&SB n = 56, ISUP 1 at TB only n = 13, ISUP 1 at SB only n = 43) C Adverse pathology findings based on ISUP 2 detecting biopsy approach (ISUP 1 at TB&SB n = 56, ISUP 1 at TB only n = 13, ISUP 1 at SB only n = 43), TB targeted biopsies, SB systematic biopsies, ISUP International Society of Urological Pathology, AP adverse pathology, RP radical prostatectomy (PPTX 877 KB) [file 345_2022_4236_MOESM1_ESM.pptx]

## Slide 1
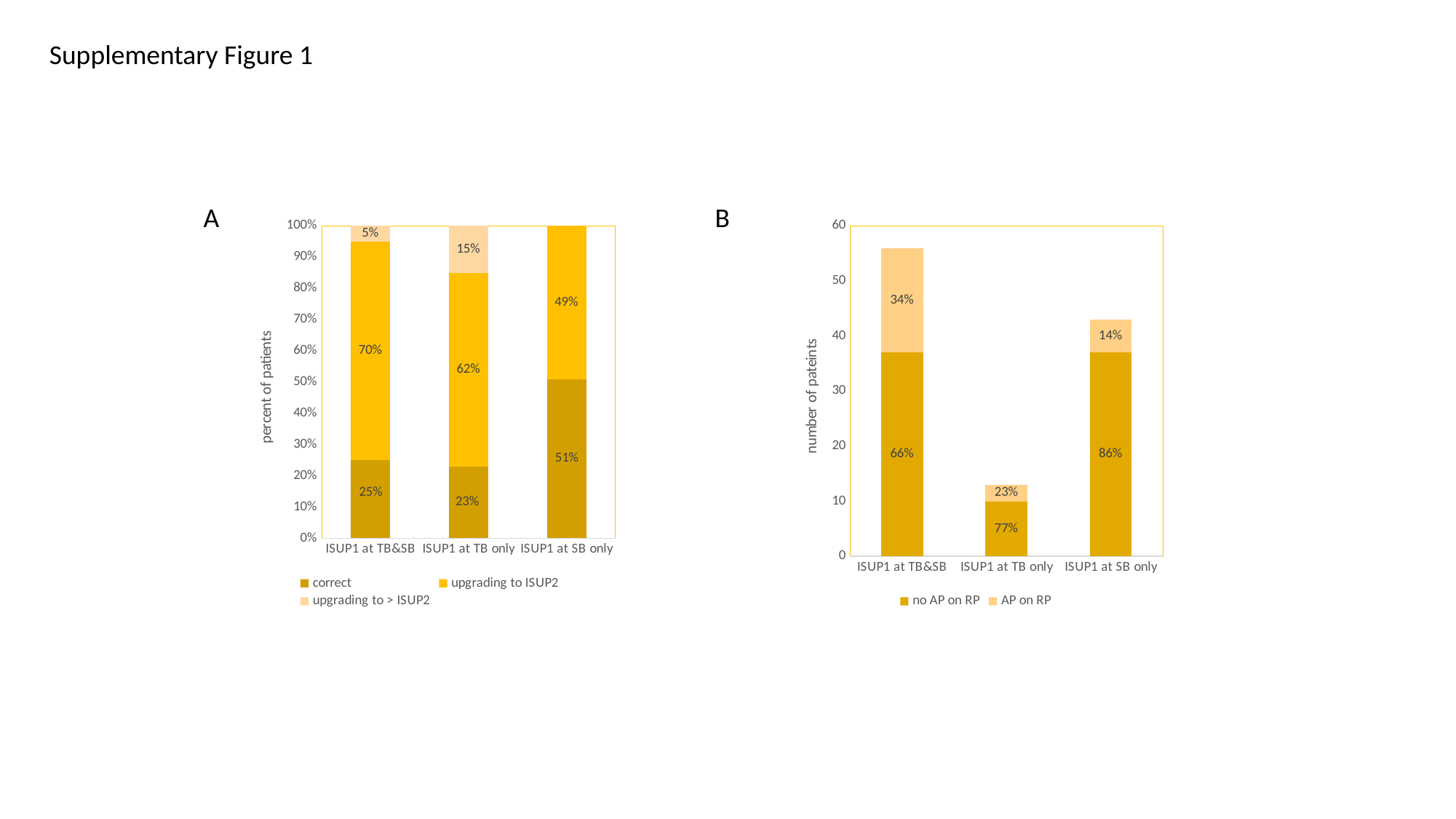

Supplementary Figure 1
A
B
### Chart
| Category | no AP on RP | AP on RP |
|---|---|---|
| ISUP1 at TB&SB | 37.0 | 19.0 |
| ISUP1 at TB only | 10.0 | 3.0 |
| ISUP1 at SB only | 37.0 | 6.0 |
### Chart
| Category | correct | upgrading to ISUP2 | upgrading to > ISUP2 |
|---|---|---|---|
| ISUP1 at TB&SB | 0.25 | 0.7 | 0.05 |
| ISUP1 at TB only | 0.23 | 0.62 | 0.15 |
| ISUP1 at SB only | 0.51 | 0.49 | 0.0 |
