# Supplementary material for: Risk and predictors of adverse pathology after radical prostatectomy in patients diagnosed with IUSP 1–2 prostate cancer at MRI-targeted biopsy: a multicenter analysis
Source: World J Urol. 2022 Dec 19;41(2):427–34. doi: 10.1007/s00345-022-04236-4 (PMC9947075; doi:10.1007/s00345-022-04236-4)
Supplement: Supplementary file 2 — Supplementary file2 ISUP upgrading from biopsy to radical prostatectomy (DOCX 15 KB) [file 345_2022_4236_MOESM2_ESM.docx]

Supplementary Table 1.

| **Low-risk PCa** | | |  |  | |  | |  | | |  |  | |  | |  |
| --- | --- | --- | --- | --- | --- | --- | --- | --- | --- | --- | --- | --- | --- | --- | --- | --- |
| ISUP Upgrading >=3 (n = 5) | | | |  | |  | |  | | |  |  | |  | |  |
| Number to small for analysis | | | |  | |  | |  | | |  |  | |  | |  |
|  |  | |  |  | |  | |  | | |  |  | |  | |  |
| AP (n = 28) | | |  |  | |  | |  | | |  |  | |  | |  |
| DRE | OR | 2.96 | | | 95%CI | | 1.18 | | - | 7.41 | | | p = | | 0.02 | |
| PSA | OR | 1.48 | | | 95%CI | | 1.14 | | - | 1.92 | | | p ≤ | | 0.01 | |
| PSA density | OR | 3016.49 | | | 95%CI | | 4.31 | | - | 2113270.3 | | | p = | | 0.02 | |
| ECE | OR | 3.55 | | | 95%CI | | 1.04 | | - | 12.09 | | | p = | | 0.04 | |
| ISUP TB | OR | 2.89 | | | 95%CI | | 1.06 | | - | 7.85 | | | p = | | 0.04 | |

| **Favorable intermediate-risk PCa** | | | | | |  | |  | | |  |  | |  | |  |
| --- | --- | --- | --- | --- | --- | --- | --- | --- | --- | --- | --- | --- | --- | --- | --- | --- |
| ISUP Upgrading >=3 (n = 101) | | | |  | |  | |  | | |  |  | |  | |  |
| Lesion vol. | OR | 1.24 | | | 95%CI | | 1.05 | | - | 1.47 | | | p = | | 0.01 | |
| % TB + | OR | 1.01 | | | 95%CI | | 1.00 | | - | 1.02 | | | p = | | 0.03 | |
| N cores SB | OR | 1.09 | | | 95%CI | | 1.03 | | - | 1.14 | | | p ≤ | | 0.01 | |
| N cores overall | OR | 1.07 | | | 95%CI | | 1.03 | | - | 1.12 | | | p ≤ | | 0.01 | |
| % overall + | OR | 1.01 | | | 95%CI | | 1.00 | | - | 1.03 | | | p = | | 0.05 | |
|  |  | |  |  | |  | |  | | |  |  | |  | |  |
| AP (n = 116) | | | |  | |  | |  | | |  |  | |  | |  |
| % TB + | OR | 1.01 | | | 95%CI | | 1.00 | | - | 1.03 | | | p ≤ | | 0.01 | |
| ISUP TB | OR | 1.49 | | | 95%CI | | 1.04 | | - | 2.14 | | | p = | | 0.03 | |
| % SB + | OR | 1.01 | | | 95%CI | | 1.00 | | - | 1.03 | | | p = | | 0.02 | |
| % overall + | OR | 1.02 | | | 95%CI | | 1.01 | |  | 1.04 | | | p ≤ | | 0.01 | |

PCa = prostate cancer, PSA = prostate specific antigen, TB = targeted biopsies, SB = systematic biopsies, ISUP = International Society of Urological Pathology, AP = adverse pathology, RP = radical prostatectomy, ECE = extraprostatic extension, DRE = digital rectal examination
