# Supplementary material for: Risk and predictors of adverse pathology after radical prostatectomy in patients diagnosed with IUSP 1–2 prostate cancer at MRI-targeted biopsy: a multicenter analysis
Source: World J Urol. 2022 Dec 19;41(2):427–34. doi: 10.1007/s00345-022-04236-4 (PMC9947075; doi:10.1007/s00345-022-04236-4)
Supplement: Supplementary file 3 — Supplementary file3 Predictors for upgrading to ISUP ≥ 3 and adverse pathology stratified by subgroups (univariate logistic regression) (DOCX 17 KB) [file 345_2022_4236_MOESM3_ESM.docx]

Table 2.

| A |  |  | **ISUP at RP** |  |  |  |  |  |
| --- | --- | --- | --- | --- | --- | --- | --- | --- |
|  |  |  | **1** | **2** | **3** | **4** | **5** | total |
| **ISUP at TB & SB** |  | **no cancer** | 0 (0%) | 0 (0%) | 0 (0%) | 0 (0%) | 0 (0%) | 0 (0%) |
|  |  | **1** | 49 (10%) | 84 (18%) | 13 (3%) | 1 (0%) | 0 (0%) | 147 (31%) |
|  |  | **2** | 9 (2%) | 232 (49%) | 76 (16%) | 7 (1%) | 4 (1%) | 328 (69%) |
|  |  | total | 58 (12%) | 316 (67%) | 89 (19%) | 8 (1%) | 4 (1%) | 475 (100%) |
|  |  |  |  |  |  |  |  |  |
|  |  |  |  |  |  |  |  |  |
| B |  |  | **ISUP at RP** |  |  |  |  |  |
|  |  |  | **1** | **2** | **3** | **4** | **5** | total |
| **ISUP at TB** |  | **no cancer** | 28 (6%) | 63 (13%) | 16 (3%) | 1 (0%) | 1 (0%) | 109 (23%) |
|  |  | **1** | 24 (5%) | 69 (15%) | 13 (3%) | 1 (0%) | 0 (0%) | 107 (23%) |
|  |  | **2** | 6 (1%) | 184 (39%) | 60 (13%) | 6 (1%) | 3 (1%) | 259 (55%) |
|  |  | total | 58 (12%) | 316 (67%) | 89 (19%) | 8 (1%) | 4 (1%) | 475 (100%) |
|  |  |  |  |  |  |  |  |  |
|  |  |  |  |  |  |  |  |  |
| C |  |  | **ISUP at RP** |  |  |  |  |  |
|  |  |  | **1** | **2** | **3** | **4** | **5** | total |
| **ISUP at SB** |  | **no cancer** | 4 (1%) | 44 (10%) | 13 (3%) | 1 (0%) | 0 (0%) | 62 (13%) |
|  |  | **1** | 47 (10%) | 109 (23%) | 16 (3%) | 0 (0%) | 1 (0%) | 173 (36%) |
|  |  | **2** | 7 (1%) | 163 (34%) | 60 (13%) | 7 (1%) | 3 (1%) | 240 (51%) |
|  |  | total | 58 (12%) | 316 (67%) | 89 (19%) | 8 (1%) | 4 (1%) | 475 (100%) |

ISUP = International Society of Urological Pathology, RP = radical prostatectomy, TB = targeted biopsies, SB = systematic biopsies
